# Human African Trypanosomiasis in Emigrant Returning to China from Gabon, 2017

**DOI:** 10.3201/eid2402.171583

**Published:** 2018-02

**Authors:** Xinyu Wang, Qiaoling Ruan, Bin Xu, Jianfei Gu, Yiyi Qian, Muxin Chen, Qin Liu, Qing Lu, Wenhong Zhang

**Affiliations:** Fudan University, Shanghai, China (X. Wang, Q. Ruan, B. Xu, J. Gu, Y. Qian, Q. Lu, W. Zhang);; Chinese Center for Disease Control and Prevention, WHO Collaborating Center for Tropical Diseases, Shanghai (M. Chen, Q. Liu)

**Keywords:** Human African trypanosomiasis, *Trypanosoma* *brucei* *gambiense*, African sleeping sickness, travel, Gabon, Shanghai, China, tsetse fly, central Africa, somnolence, fatigue, parasites, trypomastigotes, vector-borne infectious

## Abstract

Human African trypanosomiasis is endemic to parts of sub-Saharan Africa and should be considered in the differential diagnosis of patients who have visited or lived in Africa. We report a 2017 case of stage 2 *Trypanosoma brucei gambiense* disease in an emigrant who returned to China from Gabon.

Human African trypanosomiasis (HAT), or sleeping sickness, is a tsetse fly–borne parasitic disease that is endemic to parts of sub-Saharan Africa. In central and west Africa, *Trypanosoma brucei gambiense* causes the slow-progressing form of the disease, and *T. brucei rhodesiense* causes the fast-progressing form in east and southern Africa (*1*). We report a confirmed case of HAT, after a probable tsetse fly bite, in a man who returned to China from Gabon in central Africa.

A previously healthy 60-year-old man from China lived in Gabon for 8 years. He served as a seaman and traveled between Libreville and Kango to transport river sand. In July 2016, when he was working on a rural farm in Libreville, he had a painful, unidentified insect bite on his right lower limb. The bite wound developed into an indurated, erythematous, and painful skin lesion. He received antiviral and antityphoid therapy in Gabon. Although the skin lesion healed, he had intermittent fever (up to 40°C), headache, and fatigue. 

He returned to Jiangshu, China, for further treatment in June 2017. Magnetic resonance imaging (MRI) of the brain revealed temporal foci suggestive of white matter demyelination. Brain magnetic resonance angiography and electroencephalography revealed normal findings. He had daytime somnolence 2 weeks before admission to Huashan Hospital, associated with Fudan University in Shanghai, on August 30. The patient was lethargic during admission and had a temperature of 38.5°C and palpable cervical and inguinal lymph nodes. Hyperpigmentation of the right lower limb was visible. Meningeal irritation and the Babinski sign were absent.

The preliminary diagnosis was suspected HAT. We performed bone marrow puncture, which revealed a few trypomastigotes (*Trypanosoma* spp.; Figure, panel A). We also found trypanosomes in a peripheral blood smear. We sent a serum sample to the Chinese Center for Disease Control and Prevention (China CDC), which showed a positive result for the *T. brucei gambiense* antibody test. A cerebrospinal fluid (CSF) sample revealed an open pressure of 15 cm H_2_O, a leukocyte count of 9 cells/μL, a protein level of 1,412 mg/dL, and a glucose level of 1.6 mmol/L. Direct examination revealed no trypanosomes in the CSF, although next-generation sequencing identified *T. brucei gambiense* in the CSF and bone marrow (stage 2 disease). Brain MRI revealed hyperintense signal changes in the left basal ganglia, and positron emission tomography–computed tomography suggested reduced glucose metabolism (Figure, panels B, C). The World Health Organization (WHO) and China CDC helped obtain nifurtimox and eflornithine, which we administered to the patient within 48 h after the diagnosis. The patient was discharged after 10 days of treatment.

**Figure:**
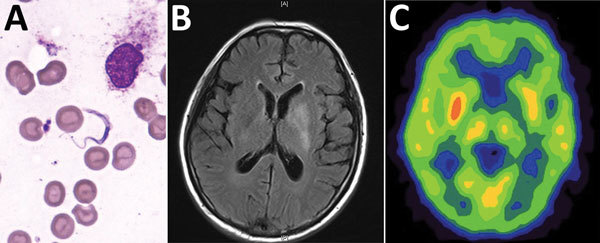
Bone marrow test results and brain imaging of a 60-year-old man who returned to China from Gabon with suspected human African trypanosomiasis. A) *Trypanosoma* spp. (later determined to be *T. brucei gambiense*) in a Giemsa-stained thin bone marrow film. Original magnification ×1,000. B) A T2-weighted fluid-attenuated inversion recovery image with hyperintense signal changes in the left basal ganglia. C) Brain positron emission tomography–computed tomography suggested reduced glucose metabolism in the left basal ganglia.

Several HAT cases had previously been imported into China. One case-patient was a 45-year-old man who worked in forests and river valleys in Gabon and was diagnosed with *T. brucei gambiense* disease by blood smear in 2014, two months after returning to China (*2*). The second case involved a woman, 41 years of age, who traveled to Tanzania and Kenya, and was diagnosed with *T. brucei rhodesiense* by blood smear in 2017, one week after returning to China. Both cases were confirmed by molecular diagnosis. Additional cases may have been imported to China, but we could not find any reports after a review of literature and available national records. 

The latest data from WHO indicate that new HAT cases have declined during the past 15 years, reaching a low of 2,184 cases in 2016 (*3*). Nevertheless, HAT cases exported from Africa have been reported in all continents. In non–HAT-endemic areas, 94 cases of HAT were reported during 2000–2010, including 26 cases of *T. brucei gambiense* disease (*4*). *T. brucei rhodesiense* disease typically involves tourists who have visited national parks and game reserves in eastern and southern Africa, whereas *T. brucei gambiense* disease is rarer and typically involves migrants and long-term emigrants (*1*). Persons in Gabon are at risk for *T. brucei gambiense* infection; 403 cases were reported during 2010–2014 (*4*). Nine exported HAT cases from Gabon have been reported, including 5 from Libreville; all these cases were *T. brucei gambiense* disease, and 6 were in immigrants from various countries (*2*,*5*–*7*).

Although HAT is uncommon in nonendemic settings, it should be included in the differential diagnosis of travelers who return from sub-Saharan Africa with fever. *T. brucei rhodesiense* disease causes acute illness and usually manifests as severe parasitemia; thus, the diagnosis is easier and quicker. In contrast, *T. brucei gambiense* disease usually has a chronic progressive course, with an estimated untreated infection duration of 2 years (*8*). Blood film review is essential for the early detection of HAT. Moreover, *T. brucei gambiense* infection can be reliably diagnosed by testing for specific antibodies, although this test is not available in most areas of China. As in this case, next-generation sequencing technologies are useful for diagnosing unknown tropical febrile illnesses (*9*). Because rapid diagnosis and treatment of HAT are essential, countries with large populations of travelers to HAT-endemic regions should maintain diagnostic tools and appropriate medication to facilitate rapid clinical management.
